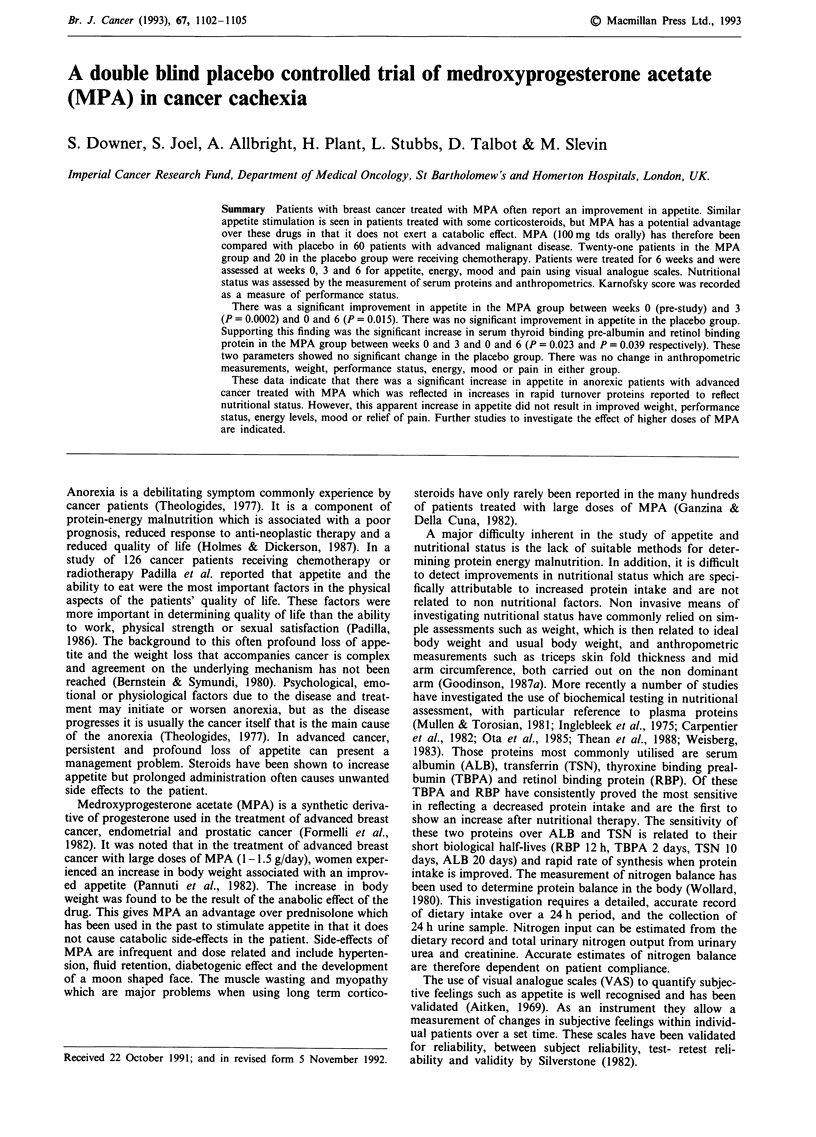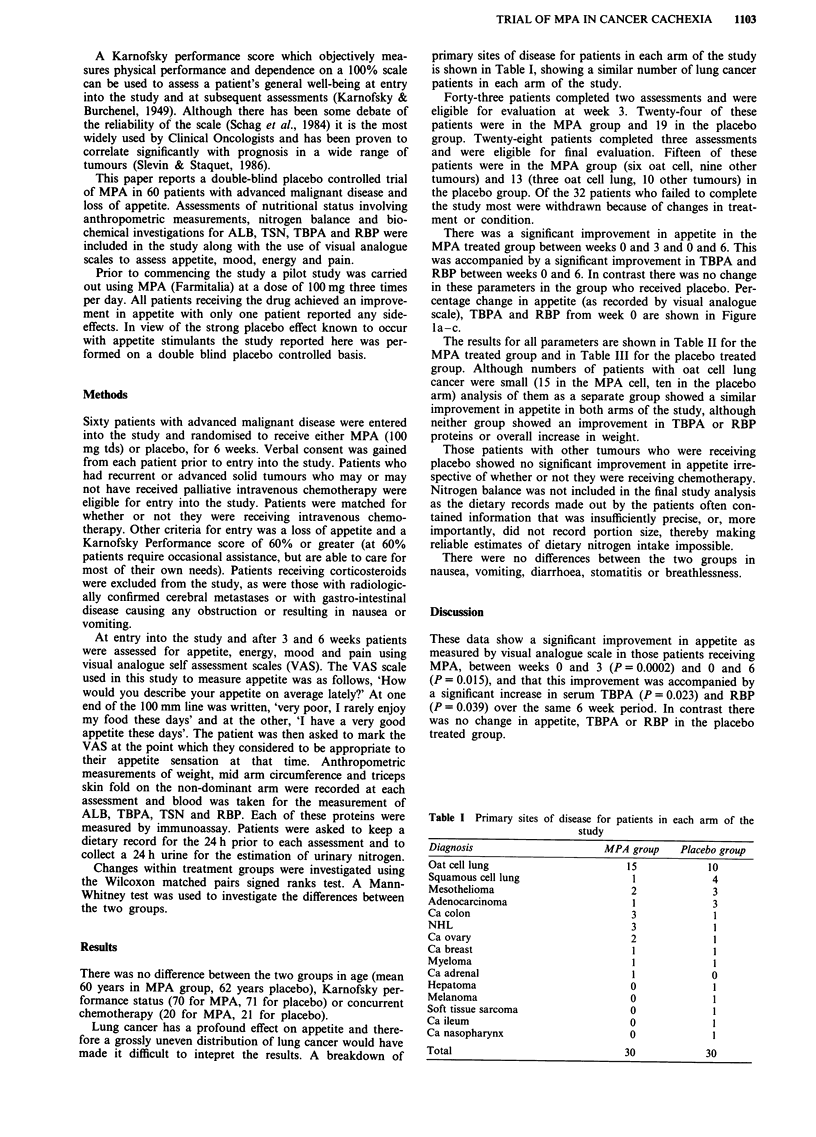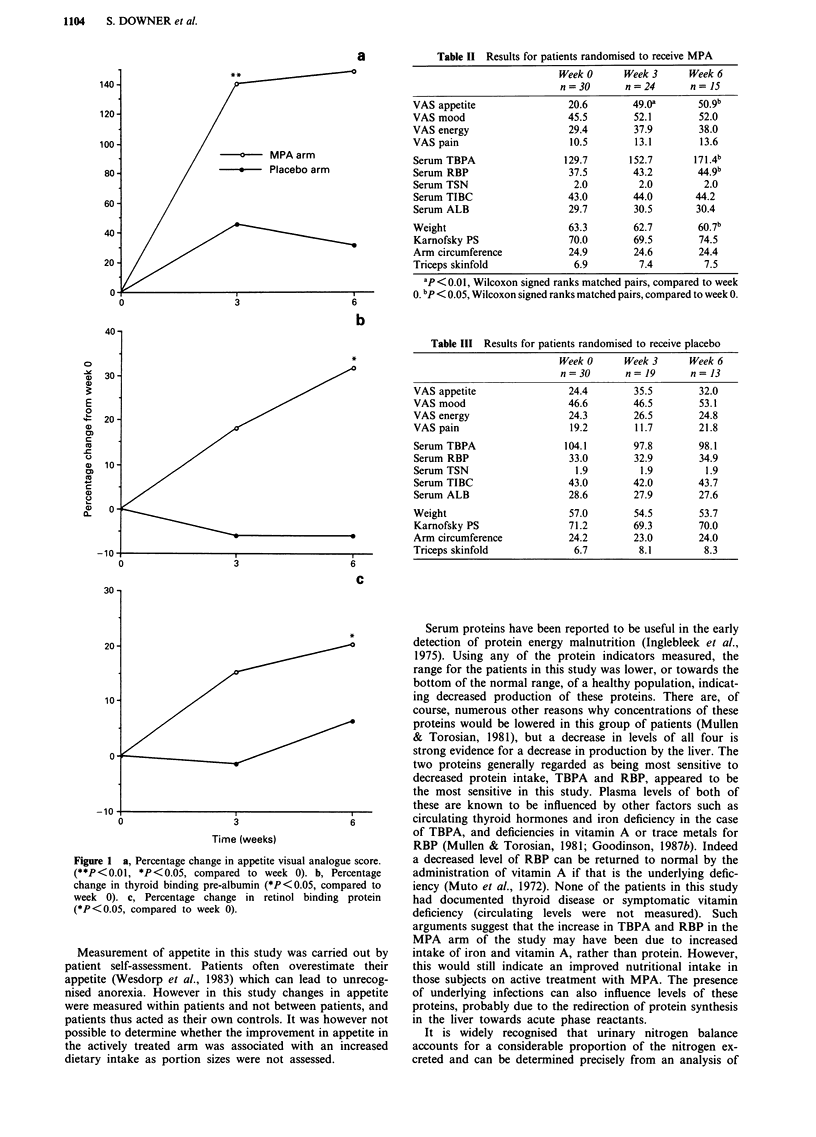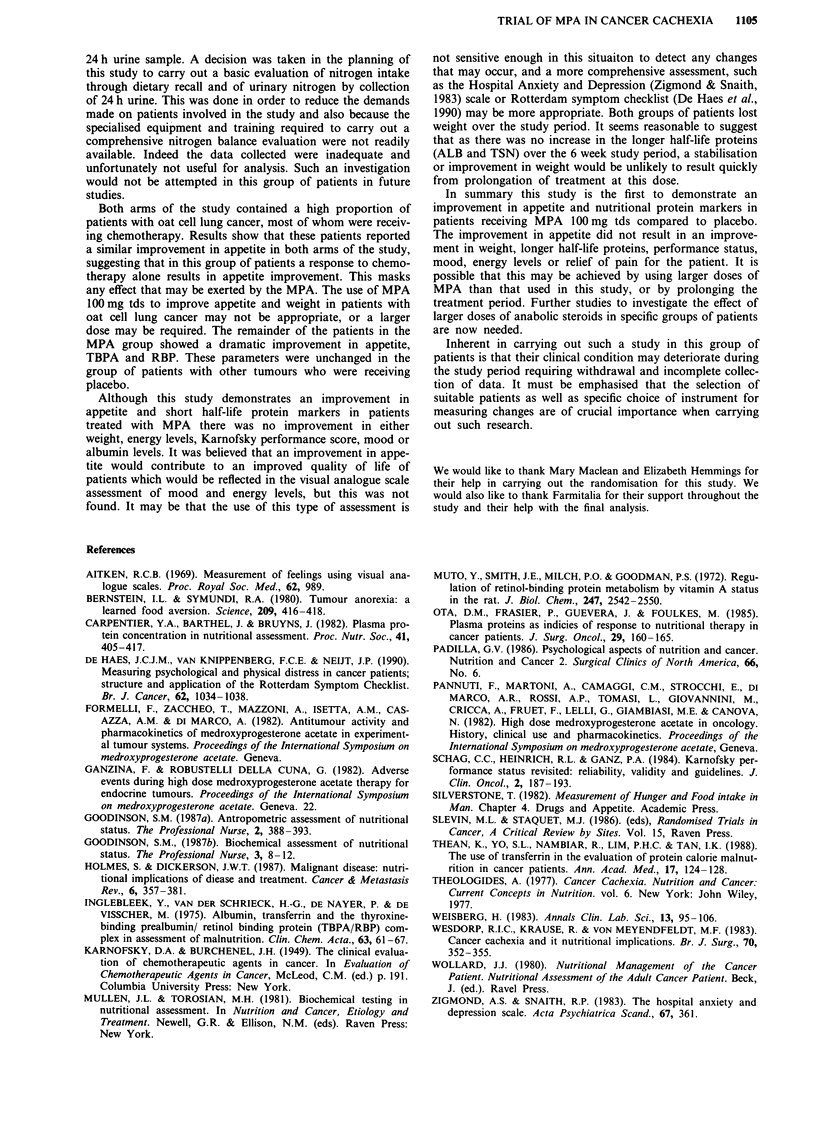# A double blind placebo controlled trial of medroxyprogesterone acetate (MPA) in cancer cachexia.

**DOI:** 10.1038/bjc.1993.202

**Published:** 1993-05

**Authors:** S. Downer, S. Joel, A. Allbright, H. Plant, L. Stubbs, D. Talbot, M. Slevin

**Affiliations:** Imperial Cancer Research Fund, Department of Medical Oncology, St Bartholomew's Hospital, London, UK.

## Abstract

Patients with breast cancer treated with MPA often report an improvement in appetite. Similar appetite stimulation is seen in patients treated with some corticosteroids, but MPA has a potential advantage over these drugs in that it does not exert a catabolic effect. MPA (100 mg tds orally) has therefore been compared with placebo in 60 patients with advanced malignant disease. Twenty-one patients in the MPA group and 20 in the placebo group were receiving chemotherapy. Patients were treated for 6 weeks and were assessed at weeks 0, 3 and 6 for appetite, energy, mood and pain using visual analogue scales. Nutritional status was assessed by the measurement of serum proteins and anthropometrics. Karnofsky score was recorded as a measure of performance status. There was a significant improvement in appetite in the MPA group between weeks 0 (pre-study) and 3 (P = 0.0002) and 0 and 6 (P = 0.015). There was no significant improvement in appetite in the placebo group. Supporting this finding was the significant increase in serum thyroid binding pre-albumin and retinol binding protein in the MPA group between weeks 0 and 3 and 0 and 6 (P = 0.023 and P = 0.039 respectively). These two parameters showed no significant change in the placebo group. There was no change in anthropometric measurements, weight, performance status, energy, mood or pain in either group. These data indicate that there was a significant increase in appetite in anorexic patients with advanced cancer treated with MPA which was reflected in increases in rapid turnover proteins reported to reflect nutritional status. However, this apparent increase in appetite did not result in improved weight, performance status, energy levels, mood or relief of pain. Further studies to investigate the effect of higher doses of MPA are indicated.


Br. J. Cancer (1993), 67, 1102–1105 © Macmillan Press Ltd., 1993

### A double blind placebo controlled trial of medroxyprogesterone acetate (MPA) in cancer cachexia

S. Downer, S. Joel, A. Allbright, H. Plant, L. Stubbs, D. Talbot & M. Slevin

*Imperial Cancer Research Fund, Department of Medical Oncology, St Bartholomew's and Homerton Hospitals, London, UK.*

**Summary** Patients with breast cancer treated with MPA often report an improvement in appetite. Similar appetite stimulation is seen in patients treated with some corticosteroids, but MPA has a potential advantage over these drugs in that it does not exert a catabolic effect. MPA (100 mg tds orally) has therefore been compared with placebo in 60 patients with advanced malignant disease. Twenty-one patients in the MPA group and 20 in the placebo group were receiving chemotherapy. Patients were treated for 6 weeks and were assessed at weeks 0, 3 and 6 for appetite, energy, mood and pain using visual analogue scales. Nutritional status was assessed by the measurement of serum proteins and anthropometrics. Karnofsky score was recorded as a measure of performance status.

There was a significant improvement in appetite in the MPA group between weeks 0 (pre-study) and 3 ($P = 0.0002$) and 0 and 6 ($P = 0.015$). There was no significant improvement in appetite in the placebo group. Supporting this finding was the significant increase in serum thyroid binding pre-albumin and retinol binding protein in the MPA group between weeks 0 and 3 and 0 and 6 ($P = 0.023$ and $P = 0.039$ respectively). These two parameters showed no significant change in the placebo group. There was no change in anthropometric measurements, weight, performance status, energy, mood or pain in either group.

These data indicate that there was a significant increase in appetite in anorexic patients with advanced cancer treated with MPA which was reflected in increases in rapid turnover proteins reported to reflect nutritional status. However, this apparent increase in appetite did not result in improved weight, performance status, energy levels, mood or relief of pain. Further studies to investigate the effect of higher doses of MPA are indicated.

Anorexia is a debilitating symptom commonly experience by cancer patients (Theologides, 1977). It is a component of protein-energy malnutrition which is associated with a poor prognosis, reduced response to anti-neoplastic therapy and a reduced quality of life (Holmes & Dickerson, 1987). In a study of 126 cancer patients receiving chemotherapy or radiotherapy Padilla *et al.* reported that appetite and the ability to eat were the most important factors in the physical aspects of the patients' quality of life. These factors were more important in determining quality of life than the ability to work, physical strength or sexual satisfaction (Padilla, 1986). The background to this often profound loss of appetite and the weight loss that accompanies cancer is complex and agreement on the underlying mechanism has not been reached (Bernstein & Symundi, 1980). Psychological, emotional or physiological factors due to the disease and treatment may initiate or worsen anorexia, but as the disease progresses it is usually the cancer itself that is the main cause of the anorexia (Theologides, 1977). In advanced cancer, persistent and profound loss of appetite can present a management problem. Steroids have been shown to increase appetite but prolonged administration often causes unwanted side effects to the patient.

Medroxyprogesterone acetate (MPA) is a synthetic derivative of progesterone used in the treatment of advanced breast cancer, endometrial and prostatic cancer (Formelli *et al.*, 1982). It was noted that in the treatment of advanced breast cancer with large doses of MPA (1 – 1.5 g/day), women experienced an increase in body weight associated with an improved appetite (Pannuti *et al.*, 1982). The increase in body weight was found to be the result of the anabolic effect of the drug. This gives MPA an advantage over prednisolone which has been used in the past to stimulate appetite in that it does not cause catabolic side-effects in the patient. Side-effects of MPA are infrequent and dose related and include hypertension, fluid retention, diabetogenic effect and the development of a moon shaped face. The muscle wasting and myopathy which are major problems when using long term corticosteroids have only rarely been reported in the many hundreds of patients treated with large doses of MPA (Ganzina & Della Cuna, 1982).

A major difficulty inherent in the study of appetite and nutritional status is the lack of suitable methods for determining protein energy malnutrition. In addition, it is difficult to detect improvements in nutritional status which are specifically attributable to increased protein intake and are not related to non nutritional factors. Non invasive means of investigating nutritional status have commonly relied on simple assessments such as weight, which is then related to ideal body weight and usual body weight, and anthropometric measurements such as triceps skin fold thickness and mid arm circumference, both carried out on the non dominant arm (Goodinson, 1987*a*). More recently a number of studies have investigated the use of biochemical testing in nutritional assessment, with particular reference to plasma proteins (Mullen & Torosian, 1981; Inglebleek *et al.*, 1975; Carpentier *et al.*, 1982; Ota *et al.*, 1985; Thean *et al.*, 1988; Weisberg, 1983). Those proteins most commonly utilised are serum albumin (ALB), transferrin (TSN), thyroxine binding prealbumin (TBPA) and retinol binding protein (RBP). Of these TBPA and RBP have consistently proved the most sensitive in reflecting a decreased protein intake and are the first to show an increase after nutritional therapy. The sensitivity of these two proteins over ALB and TSN is related to their short biological half-lives (RBP 12 h, TBPA 2 days, TSN 10 days, ALB 20 days) and rapid rate of synthesis when protein intake is improved. The measurement of nitrogen balance has been used to determine protein balance in the body (Wollard, 1980). This investigation requires a detailed, accurate record of dietary intake over a 24 h period, and the collection of 24 h urine sample. Nitrogen input can be estimated from the dietary record and total urinary nitrogen output from urinary urea and creatinine. Accurate estimates of nitrogen balance are therefore dependent on patient compliance.

The use of visual analogue scales (VAS) to quantify subjective feelings such as appetite is well recognised and has been validated (Aitken, 1969). As an instrument they allow a measurement of changes in subjective feelings within individual patients over a set time. These scales have been validated for reliability, between subject reliability, test- retest reliability and validity by Silverstone (1982).

Received 22 October 1991; and in revised form 5 November 1992.

A Karnofsky performance score which objectively measures physical performance and dependence on a 100% scale can be used to assess a patient's general well-being at entry into the study and at subsequent assessments (Karnofsky & Burchenel, 1949). Although there has been some debate of the reliability of the scale (Schag *et al.*, 1984) it is the most widely used by Clinical Oncologists and has been proven to correlate significantly with prognosis in a wide range of tumours (Slevin & Staquet, 1986).

This paper reports a double-blind placebo controlled trial of MPA in 60 patients with advanced malignant disease and loss of appetite. Assessments of nutritional status involving anthropometric measurements, nitrogen balance and biochemical investigations for ALB, TSN, TBPA and RBP were included in the study along with the use of visual analogue scales to assess appetite, mood, energy and pain.

Prior to commencing the study a pilot study was carried out using MPA (Farmitalia) at a dose of 100 mg three times per day. All patients receiving the drug achieved an improvement in appetite with only one patient reported any side-effects. In view of the strong placebo effect known to occur with appetite stimulants the study reported here was performed on a double blind placebo controlled basis.

### Methods

Sixty patients with advanced malignant disease were entered into the study and randomised to receive either MPA (100 mg tds) or placebo, for 6 weeks. Verbal consent was gained from each patient prior to entry into the study. Patients who had recurrent or advanced solid tumours who may or may not have received palliative intravenous chemotherapy were eligible for entry into the study. Patients were matched for whether or not they were receiving intravenous chemotherapy. Other criteria for entry was a loss of appetite and a Karnofsky Performance score of 60% or greater (at 60% patients require occasional assistance, but are able to care for most of their own needs). Patients receiving corticosteroids were excluded from the study, as were those with radiologically confirmed cerebral metastases or with gastro-intestinal disease causing any obstruction or resulting in nausea or vomiting.

At entry into the study and after 3 and 6 weeks patients were assessed for appetite, energy, mood and pain using visual analogue self assessment scales (VAS). The VAS scale used in this study to measure appetite was as follows, 'How would you describe your appetite on average lately?' At one end of the 100 mm line was written, 'very poor, I rarely enjoy my food these days' and at the other, 'I have a very good appetite these days'. The patient was then asked to mark the VAS at the point which they considered to be appropriate to their appetite sensation at that time. Anthropometric measurements of weight, mid arm circumference and triceps skin fold on the non-dominant arm were recorded at each assessment and blood was taken for the measurement of ALB, TBPA, TSN and RBP. Each of these proteins were measured by immunoassay. Patients were asked to keep a dietary record for the 24 h prior to each assessment and to collect a 24 h urine for the estimation of urinary nitrogen.

Changes within treatment groups were investigated using the Wilcoxon matched pairs signed ranks test. A Mann-Whitney test was used to investigate the differences between the two groups.

### Results

There was no difference between the two groups in age (mean 60 years in MPA group, 62 years placebo), Karnofsky performance status (70 for MPA, 71 for placebo) or concurrent chemotherapy (20 for MPA, 21 for placebo).

Lung cancer has a profound effect on appetite and therefore a grossly uneven distribution of lung cancer would have made it difficult to intepret the results. A breakdown of primary sites of disease for patients in each arm of the study is shown in Table I, showing a similar number of lung cancer patients in each arm of the study.

Forty-three patients completed two assessments and were eligible for evaluation at week 3. Twenty-four of these patients were in the MPA group and 19 in the placebo group. Twenty-eight patients completed three assessments and were eligible for final evaluation. Fifteen of these patients were in the MPA group (six oat cell, nine other tumours) and 13 (three oat cell lung, 10 other tumours) in the placebo group. Of the 32 patients who failed to complete the study most were withdrawn because of changes in treatment or condition.

There was a significant improvement in appetite in the MPA treated group between weeks 0 and 3 and 0 and 6. This was accompanied by a significant improvement in TBPA and RBP between weeks 0 and 6. In contrast there was no change in these parameters in the group who received placebo. Percentage change in appetite (as recorded by visual analogue scale), TBPA and RBP from week 0 are shown in Figure 1a–c.

The results for all parameters are shown in Table II for the MPA treated group and in Table III for the placebo treated group. Although numbers of patients with oat cell lung cancer were small (15 in the MPA cell, ten in the placebo arm) analysis of them as a separate group showed a similar improvement in appetite in both arms of the study, although neither group showed an improvement in TBPA or RBP proteins or overall increase in weight.

Those patients with other tumours who were receiving placebo showed no significant improvement in appetite irrespective of whether or not they were receiving chemotherapy. Nitrogen balance was not included in the final study analysis as the dietary records made out by the patients often contained information that was insufficiently precise, or, more importantly, did not record portion size, thereby making reliable estimates of dietary nitrogen intake impossible.

There were no differences between the two groups in nausea, vomiting, diarrhoea, stomatitis or breathlessness.

### Discussion

These data show a significant improvement in appetite as measured by visual analogue scale in those patients receiving MPA, between weeks 0 and 3 ($P = 0.0002$) and 0 and 6 ($P = 0.015$), and that this improvement was accompanied by a significant increase in serum TBPA ($P = 0.023$) and RBP ($P = 0.039$) over the same 6 week period. In contrast there was no change in appetite, TBPA or RBP in the placebo treated group.

**Table I** Primary sites of disease for patients in each arm of the study

| *Diagnosis* | *MPA group* | *Placebo group* |
|---|---|---|
| Oat cell lung | 15 | 10 |
| Squamous cell lung | 1 | 4 |
| Mesothelioma | 2 | 3 |
| Adenocarcinoma | 1 | 3 |
| Ca colon | 3 | 1 |
| NHL | 3 | 1 |
| Ca ovary | 2 | 1 |
| Ca breast | 1 | 1 |
| Myeloma | 1 | 1 |
| Ca adrenal | 1 | 0 |
| Hepatoma | 0 | 1 |
| Melanoma | 0 | 1 |
| Soft tissue sarcoma | 0 | 1 |
| Ca ileum | 0 | 1 |
| Ca nasopharynx | 0 | 1 |
| Total | 30 | 30 |

**Figure 1** **a**, Percentage change in appetite visual analogue score. (**$P<0.01$, *$P<0.05$, compared to week 0). **b**, Percentage change in thyroid binding pre-albumin (*$P<0.05$, compared to week 0). **c**, Percentage change in retinol binding protein (*$P<0.05$, compared to week 0).

Measurement of appetite in this study was carried out by patient self-assessment. Patients often overestimate their appetite (Wesdorp *et al.*, 1983) which can lead to unrecognised anorexia. However in this study changes in appetite were measured within patients and not between patients, and patients thus acted as their own controls. It was however not possible to determine whether the improvement in appetite in the actively treated arm was associated with an increased dietary intake as portion sizes were not assessed.

**Table II** Results for patients randomised to receive MPA

| | *Week 0* $n=30$ | *Week 3* $n=24$ | *Week 6* $n=15$ |
|---|---|---|---|
| VAS appetite | 20.6 | 49.0[a] | 50.9[b] |
| VAS mood | 45.5 | 52.1 | 52.0 |
| VAS energy | 29.4 | 37.9 | 38.0 |
| VAS pain | 10.5 | 13.1 | 13.6 |
| Serum TBPA | 129.7 | 152.7 | 171.4[b] |
| Serum RBP | 37.5 | 43.2 | 44.9[b] |
| Serum TSN | 2.0 | 2.0 | 2.0 |
| Serum TIBC | 43.0 | 44.0 | 44.2 |
| Serum ALB | 29.7 | 30.5 | 30.4 |
| Weight | 63.3 | 62.7 | 60.7[b] |
| Karnofsky PS | 70.0 | 69.5 | 74.5 |
| Arm circumference | 24.9 | 24.6 | 24.4 |
| Triceps skinfold | 6.9 | 7.4 | 7.5 |

[a]$P<0.01$, Wilcoxon signed ranks matched pairs, compared to week 0. [b]$P<0.05$, Wilcoxon signed ranks matched pairs, compared to week 0.

**Table III** Results for patients randomised to receive placebo

| | *Week 0* $n=30$ | *Week 3* $n=19$ | *Week 6* $n=13$ |
|---|---|---|---|
| VAS appetite | 24.4 | 35.5 | 32.0 |
| VAS mood | 46.6 | 46.5 | 53.1 |
| VAS energy | 24.3 | 26.5 | 24.8 |
| VAS pain | 19.2 | 11.7 | 21.8 |
| Serum TBPA | 104.1 | 97.8 | 98.1 |
| Serum RBP | 33.0 | 32.9 | 34.9 |
| Serum TSN | 1.9 | 1.9 | 1.9 |
| Serum TIBC | 43.0 | 42.0 | 43.7 |
| Serum ALB | 28.6 | 27.9 | 27.6 |
| Weight | 57.0 | 54.5 | 53.7 |
| Karnofsky PS | 71.2 | 69.3 | 70.0 |
| Arm circumference | 24.2 | 23.0 | 24.0 |
| Triceps skinfold | 6.7 | 8.1 | 8.3 |

Serum proteins have been reported to be useful in the early detection of protein energy malnutrition (Inglebleek *et al.*, 1975). Using any of the protein indicators measured, the range for the patients in this study was lower, or towards the bottom of the normal range, of a healthy population, indicating decreased production of these proteins. There are, of course, numerous other reasons why concentrations of these proteins would be lowered in this group of patients (Mullen & Torosian, 1981), but a decrease in levels of all four is strong evidence for a decrease in production by the liver. The two proteins generally regarded as being most sensitive to decreased protein intake, TBPA and RBP, appeared to be the most sensitive in this study. Plasma levels of both of these are known to be influenced by other factors such as circulating thyroid hormones and iron deficiency in the case of TBPA, and deficiencies in vitamin A or trace metals for RBP (Mullen & Torosian, 1981; Goodinson, 1987*b*). Indeed a decreased level of RBP can be returned to normal by the administration of vitamin A if that is the underlying deficiency (Muto *et al.*, 1972). None of the patients in this study had documented thyroid disease or symptomatic vitamin deficiency (circulating levels were not measured). Such arguments suggest that the increase in TBPA and RBP in the MPA arm of the study may have been due to increased intake of iron and vitamin A, rather than protein. However, this would still indicate an improved nutritional intake in those subjects on active treatment with MPA. The presence of underlying infections can also influence levels of these proteins, probably due to the redirection of protein synthesis in the liver towards acute phase reactants.

It is widely recognised that urinary nitrogen balance accounts for a considerable proportion of the nitrogen excreted and can be determined precisely from an analysis of

24 h urine sample. A decision was taken in the planning of this study to carry out a basic evaluation of nitrogen intake through dietary recall and of urinary nitrogen by collection of 24 h urine. This was done in order to reduce the demands made on patients involved in the study and also because the specialised equipment and training required to carry out a comprehensive nitrogen balance evaluation were not readily available. Indeed the data collected were inadequate and unfortunately not useful for analysis. Such an investigation would not be attempted in this group of patients in future studies.

Both arms of the study contained a high proportion of patients with oat cell lung cancer, most of whom were receiving chemotherapy. Results show that these patients reported a similar improvement in appetite in both arms of the study, suggesting that in this group of patients a response to chemotherapy alone results in appetite improvement. This masks any effect that may be exerted by the MPA. The use of MPA 100 mg tds to improve appetite and weight in patients with oat cell lung cancer may not be appropriate, or a larger dose may be required. The remainder of the patients in the MPA group showed a dramatic improvement in appetite, TBPA and RBP. These parameters were unchanged in the group of patients with other tumours who were receiving placebo.

Although this study demonstrates an improvement in appetite and short half-life protein markers in patients treated with MPA there was no improvement in either weight, energy levels, Karnofsky performance score, mood or albumin levels. It was believed that an improvement in appetite would contribute to an improved quality of life of patients which would be reflected in the visual analogue scale assessment of mood and energy levels, but this was not found. It may be that the use of this type of assessment is not sensitive enough in this situaiton to detect any changes that may occur, and a more comprehensive assessment, such as the Hospital Anxiety and Depression (Zigmond & Snaith, 1983) scale or Rotterdam symptom checklist (De Haes *et al.*, 1990) may be more appropriate. Both groups of patients lost weight over the study period. It seems reasonable to suggest that as there was no increase in the longer half-life proteins (ALB and TSN) over the 6 week study period, a stabilisation or improvement in weight would be unlikely to result quickly from prolongation of treatment at this dose.

In summary this study is the first to demonstrate an improvement in appetite and nutritional protein markers in patients receiving MPA 100 mg tds compared to placebo. The improvement in appetite did not result in an improvement in weight, longer half-life proteins, performance status, mood, energy levels or relief of pain for the patient. It is possible that this may be achieved by using larger doses of MPA than that used in this study, or by prolonging the treatment period. Further studies to investigate the effect of larger doses of anabolic steroids in specific groups of patients are now needed.

Inherent in carrying out such a study in this group of patients is that their clinical condition may deteriorate during the study period requiring withdrawal and incomplete collection of data. It must be emphasised that the selection of suitable patients as well as specific choice of instrument for measuring changes are of crucial importance when carrying out such research.

We would like to thank Mary Maclean and Elizabeth Hemmings for their help in carrying out the randomisation for this study. We would also like to thank Farmitalia for their support throughout the study and their help with the final analysis.

**References**

AITKEN, R.C.B. (1969). Measurement of feelings using visual analogue scales. *Proc. Royal Soc. Med.*, **62**, 989.

BERNSTEIN, I.L. & SYMUNDI, R.A. (1980). Tumour anorexia: a learned food aversion. *Science*, **209**, 416–418.

CARPENTIER, Y.A., BARTHEL, J. & BRUYNS, J. (1982). Plasma protein concentration in nutritional assessment. *Proc. Nutr. Soc.*, **41**, 405–417.

DE HAES, J.C.J.M., VAN KNIPPENBERG, F.C.E. & NEIJT, J.P. (1990). Measuring psychological and physical distress in cancer patients; structure and application of the Rotterdam Symptom Checklist. *Br. J. Cancer*, **62**, 1034–1038.

FORMELLI, F., ZACCHEO, T., MAZZONI, A., ISETTA, A.M., CASAZZA, A.M. & DI MARCO, A. (1982). Antitumour activity and pharmacokinetics of medroxyprogesterone acetate in experimental tumour systems. *Proceedings of the International Symposium on medroxyprogesterone acetate*. Geneva.

GANZINA, F. & ROBUSTELLI DELLA CUNA, G. (1982). Adverse events during high dose medroxyprogesterone acetate therapy for endocrine tumours. *Proceedings of the International Symposium on medroxyprogesterone acetate*. Geneva. 22.

GOODINSON, S.M. (1987*a*). Antropometric assessment of nutritional status. *The Professional Nurse*, **2**, 388–393.

GOODINSON, S.M., (1987*b*). Biochemical assessment of nutritional status. *The Professional Nurse*, **3**, 8–12.

HOLMES, S. & DICKERSON, J.W.T. (1987). Malignant disease: nutritional implications of diease and treatment. *Cancer & Metastasis Rev.*, **6**, 357–381.

INGLEBLEEK, Y., VAN DER SCHRIECK, H.-G., DE NAYER, P. & DE VISSCHER, M. (1975). Albumin, transferrin and the thyroxine-binding prealbumin/ retinol binding protein (TBPA/RBP) complex in assessment of malnutrition. *Clin. Chem. Acta.*, **63**, 61–67.

KARNOFSKY, D.A. & BURCHENEL, J.H. (1949). The clinical evaluation of chemotherapeutic agents in cancer. In *Evaluation of Chemotherapeutic Agents in Cancer*, McLeod, C.M. (ed.) p. 191. Columbia University Press: New York.

MULLEN, J.L. & TOROSIAN, M.H. (1981). Biochemical testing in nutritional assessment. In *Nutrition and Cancer, Etiology and Treatment*. Newell, G.R. & Ellison, N.M. (eds). Raven Press: New York.

MUTO, Y., SMITH, J.E., MILCH, P.O. & GOODMAN, P.S. (1972). Regulation of retinol-binding protein metabolism by vitamin A status in the rat. *J. Biol. Chem.*, **247**, 2542–2550.

OTA, D.M., FRASIER, P., GUEVERA, J. & FOULKES, M. (1985). Plasma proteins as indicies of response to nutritional therapy in cancer patients. *J. Surg. Oncol.*, **29**, 160–165.

PADILLA, G.V. (1986). Psychological aspects of nutrition and cancer. Nutrition and Cancer 2. *Surgical Clinics of North America*, **66**, No. 6.

PANNUTI, F., MARTONI, A., CAMAGGI, C.M., STROCCHI, E., DI MARCO, A.R., ROSSI, A.P., TOMASI, L., GIOVANNINI, M., CRICCA, A., FRUET, F., LELLI, G., GIAMBIASI, M.E. & CANOVA, N. (1982). High dose medroxyprogesterone acetate in oncology. History, clinical use and pharmacokinetics. *Proceedings of the International Symposium on medroxyprogesterone acetate*, Geneva.

SCHAG, C.C., HEINRICH, R.L. & GANZ, P.A. (1984). Karnofsky performance status revisited: reliability, validity and guidelines. *J. Clin. Oncol.*, **2**, 187–193.

SILVERSTONE, T. (1982). *Measurement of Hunger and Food intake in Man*. Chapter 4. Drugs and Appetite. Academic Press.

SLEVIN, M.L. & STAQUET, M.J. (1986). (eds), *Randomised Trials in Cancer, A Critical Review by Sites*. Vol. 15, Raven Press.

THEAN, K., YO, S.L., NAMBIAR, R., LIM, P.H.C. & TAN, I.K. (1988). The use of transferrin in the evaluation of protein calorie malnutrition in cancer patients. *Ann. Acad. Med.*, **17**, 124–128.

THEOLOGIDES, A. (1977). *Cancer Cachexia. Nutrition and Cancer: Current Concepts in Nutrition*. vol. 6. New York: John Wiley, 1977.

WEISBERG, H. (1983). *Annals Clin. Lab. Sci.*, **13**, 95–106.

WESDORP, R.I.C., KRAUSE, R. & VON MEYENDFELDT, M.F. (1983). Cancer cachexia and it nutritional implications. *Br. J. Surg.*, **70**, 352–355.

WOLLARD, J.J. (1980). *Nutritional Management of the Cancer Patient. Nutritional Assessment of the Adult Cancer Patient*. Beck, J. (ed.). Ravel Press.

ZIGMOND, A.S. & SNAITH, R.P. (1983). The hospital anxiety and depression scale. *Acta Psychiatrica Scand.*, **67**, 361.